# Supplementary material for: Suppressing Pyroptosis Augments Post-Transplant Survival of Stem Cells and Cardiac Function Following Ischemic Injury
Source: Int J Mol Sci. 2021 Jul 26;22(15):7946. doi: 10.3390/ijms22157946 (PMC8348609; doi:10.3390/ijms22157946)
Supplement: Supplementary file 1 [file ijms-22-07946-s001.zip › ijms-1251838-supplementary.pdf]

Suppressing pyroptosis augments post-transplant survival of stem cells and cardiac function following ischemic injury

Chang Youn Lee,[1,†] Seahyoung Lee,[2,†] Seongtae Jeong[2], Jiyun Lee,[3] Hyang-Hee Seo,[3] Sunhye Shin,[1] Jun-Hee Park,[1] Byeong-Wook Song,[2] Il-Kwon Kim,[2] Jung-Won Choi,[2] Sang Woo Kim,[2] Gyoonhee Han,[1] Soyeon Lim,[2,*] and Ki-Chul Hwang[2,*]

1 Department of Integrated Omics for Biomedical Sciences, Yonsei University, Seoul 03722, Korea; cylee083@gmail.com (C.Y.L.); ssh5043@naver.com (S.S.); yrigmb@nate.com (J-H.P.); gyoonhee@yonsei.ac.kr (G.H.)

2 Institute for Bio-Medical Convergence, College of Medicine, Catholic Kwandong University, Gangneung-Si 25601, Korea; sam1017@ish.ac.kr (S.L.); 91seongtae@gmail.com (S.J.); songbw@gmail.com (B.-W.S.); ilkwonkim@ish.ac.kr (I.-K. K.); jungwonjian@gmail.com (J.-W.C.); doctor7408@gmail.com (S.W.K.)

3 Brain Korea 21 PLUS Project for Medical Science, Yonsei University, Seoul 03722, Korea; jylee12334@gmail.com (J.L.); seohyanghee@gmail.com (H.-H.S.)

* Correspondence: slim724@gmail.com (S.L.); kchwang@cku.ac.kr (K.-C.H.)

† These authors contributed equally to this work.

Supplementary Materials and Methods

Primary culture of neonatal cardiomyocytes and cardiac fibroblast

H9c2 cells, myoblasts derived from the ventricle of embryonic BD1X rat heart (ATCC, USA), were maintained at 37 °C in a humidified atmosphere containing 5 % CO_2. Culture media was replaced every 3 days using Dulbecco's modified Eagle's medium (DMEM, Gibco) containing 10 % fetal bovine serum (FBS; Gibco), 100 U/mL penicillin (Gibco), and 100 μg/mL streptomycin (Gibco). For isolation of neonatal rat cardiac fibroblasts (CF), CFs were prepared according to the previously published method [1]. Fibroblasts were cultured with DMEM containing 10 % FBS in a CO^2 incubator at 37 °C and then passages 2-3 were used for experiments.

IL-1β Enzyme-linked Immunosorbent Assay (ELISA)

To prepare media for IL-1β ELISA, the cells were transfected with IL-1β siRNA (50 nM) for 24 h and then stimulated with LPS/IFN-ɣ for another 24 h. To collect media containing secreted IL-1β, LPS/IFN-ɣ stimulated Raw264.7 cells were washed with serum free-DMEM and then cultured in fresh serum free-DMEM for 24 h. The culture media were collected and centrifuged at 2000 x g for 10 min to collect supernatant. The amount of IL-1β in media was determined by using a mouse IL-1 beta ELISA kit (BMS6002. Invitrogen). For estimation of secreted IL-1β in media of hASCs, human IL-1 beta ELISA kit (BMS224-2, Invitrogen) was conducted according to manufacturer's protocol.

Reference

1. Lim, S.; Chang, W.; Cha, M.J.; Song, B.W.; Ham, O.; Lee, S.Y.; Lee, C.; Park, J.H.; Lee, S.K.; Jang, Y., et al. PLCdelta1 protein rescues ischemia-reperfused heart by the regulation of calcium homeostasis. *Mol Ther* **2014**, *22*, 1110-1121, doi:10.1038/mt.2014.46.

Supplementary Figures and Figure legends

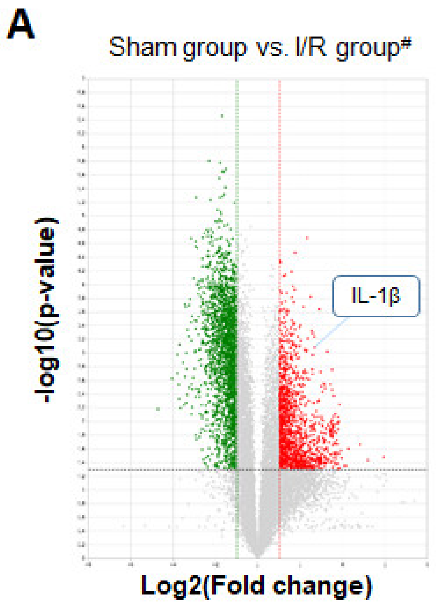

B

#	Process	n
1	# of total probes	41,078
2	# of DEG probes in (1)	3486
3	# of Inflammatory response in (1) *	84
4	# of pyroptosis in (1) **	3
5	# of intersection (3) and (4)	2

Gene	Fold Change	p-value
IL-1β	6.372	0.001
NLRC4	3.271	0.019

Figure S1. Analysis of DEG (Differentially Expressed Gene) heart with ischemia/reperfusion injury

The DEG analysis was conducted to analyze total RNA sequencing data for the identification of genes significantly changed due to ischemic injury. (A) The differences in gene expression between the I/R and sham groups were represented by a volcano plot. # I/R group: after 1days of I/R (B) Candidate genes were selected based on the DEG analysis and gene lists arranged by selection criteria indicated with * and **. n = 3 for each group. DEG; Differentially expressed gene, *Inflammatory response gene list from GO:0006954, **Pyroptosis gene list from GO:0070269.

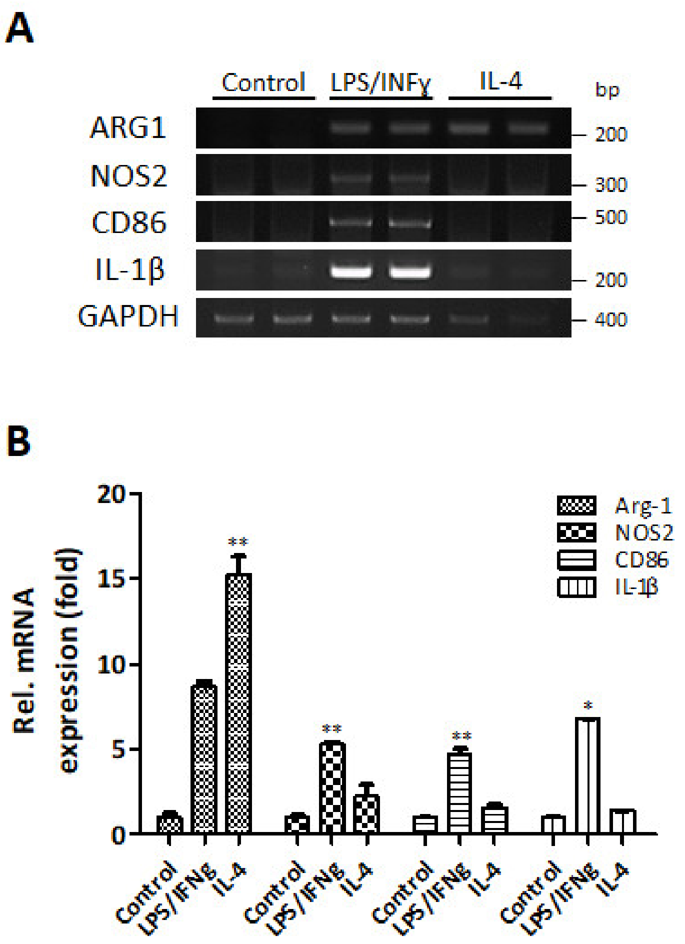

Figure S2. IL-1β expression is increased in M1-like macrophages.

(A) RAW264.7 cells were incubated with LPS/IFNɣ (M1 macrophage inducers) and IL-4 (M2 macrophage inducer) to induce differentiation into M1 and M2 macrophages. RT-PCR was performed to determine macrophage polarization. (B) ARG, NOS2, CD86, and IL-1β expression was determined by qRT-PCR and normalized to GAPDH. $^{\#}p < 0.05$ vs. Control, $^{**}p < 0.01$ vs. Control, $^{*}p < 0.001$ vs. Control. n=3.

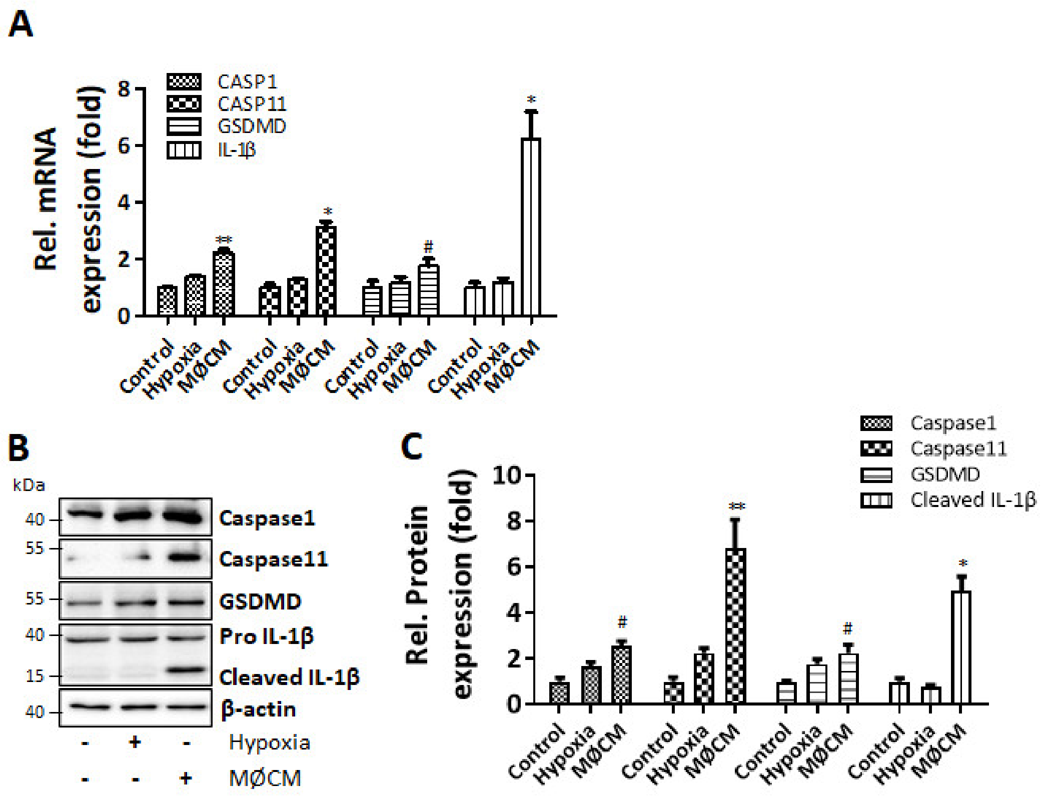

Figure S3. Increased pyroptosis-related gene and protein levels in cardiomyocytes cultured with macrophage-CM

Cardiomyocytes (H9c2) were cultured for 24 h with macrophage-CM. Pyroptosis-related genes and proteins were detected by qRT-PCR. (A) and western blot analysis (B,C). $^{\#}p < 0.05$ vs. Control, $^{**}p < 0.01$ vs. Control, $^{*}p < 0.001$ vs. Control. n=3.

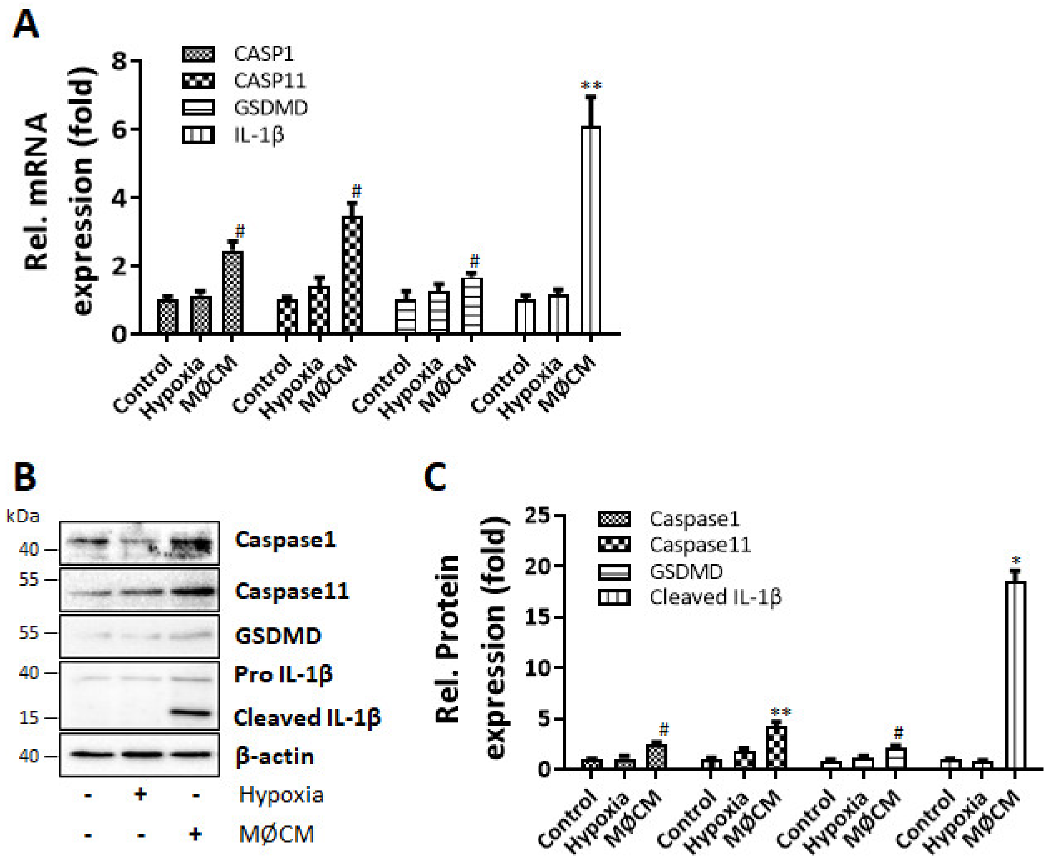

Figure S4. Increased pyroptosis-related mRNA and protein levels in cardiac fibroblasts cultured with M1 macrophage-CM

Cardiac fibroblasts were cultured for 24 h under hypoxic conditions or with M1 macrophage-CM. Pyroptosis-related mRNA and protein levels were measured by (A) qRT-PCR and (B,C) western blotting, respectively. $^{\#}p < 0.05$ vs. Control, $^{**}p < 0.01$ vs. Control, $^{*}p < 0.001$ vs. Control. n=3

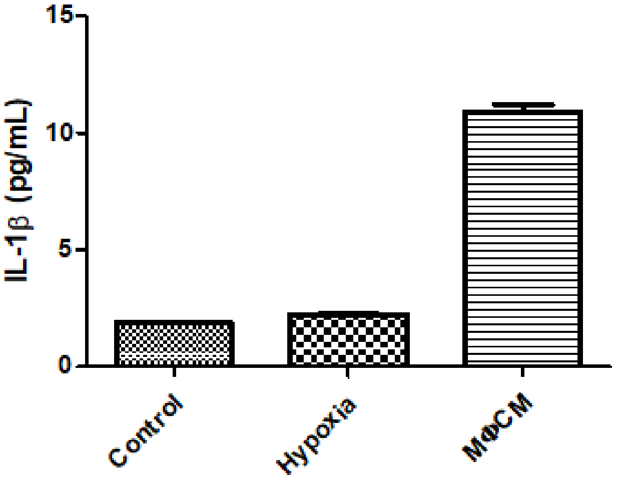

Figure S5. IL-1β secretion in ASCs stimulated with M1 macrophage CM.

hASCs were incubated with M1-like macrophage-CM (MØCM) for 24 h. The amount of IL-1β in media was determined by using an IL-1β ELISA kit. n=2.

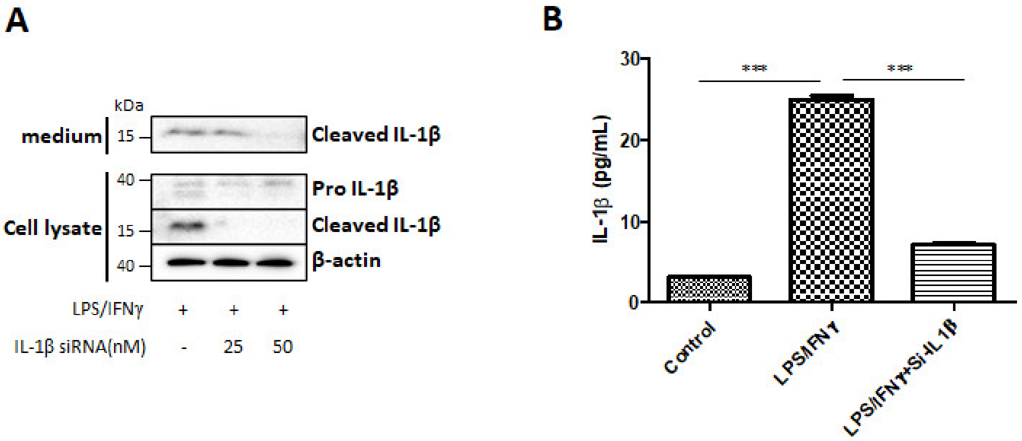

Figure S6. Effect of transient IL-1β knockdown on the expression/secretion of IL-1β protein in macrophage

Different doses of IL-1β siRNA were delivered to macrophages prior to LPS/IFN-ɣ treatment. (A) IL-1β expression in culture media and cell lysate were detected by western blotting. (B) The amount of IL-1β in culture media was determined by ELISA. *p < 0.001. n=3.

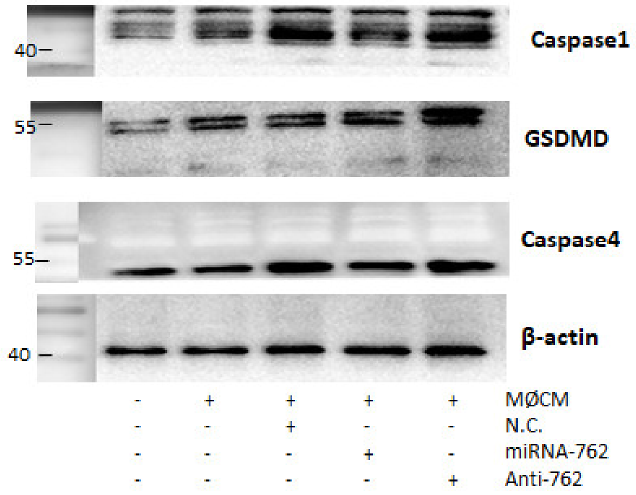

Figure S7. Pyroptosis related protein levels

Protein levels of caspase 1, GSDMD, and caspase 4 were observed in hASCs treated with miRNA-762 mimic (miRNA-762), negative control (N.C.) or anti-miRNA-762 (Anti-762).
